# Do elections matter for private-sector healthcare management in Brazil? An analysis of municipal health policy

**DOI:** 10.1186/s12913-017-2427-5

**Published:** 2017-07-12

**Authors:** Alecia J. McGregor, Carlos Eduardo Siqueira, Alan M. Zaslavsky, Robert J. Blendon

**Affiliations:** 1 Department of Community Health, Tufts University, 574 Boston Ave, Suite 208, Medford, MA 02155 USA; 2 Community Development and Planning, University of Massachusetts, Wheatley Hall, 4th Floor, 144-16, Boston, MA 02125 USA; 3 Health Care Policy, Harvard Medical School, 180 Longwood Avenue, Boston, MA 02115-5899 USA; 4 Department of Health Policy and Management, Harvard School of Public Health, 677 Huntington Ave, Boston, MA 02115 USA

**Keywords:** Health systems, Brazil, Unified health system, Sus, Private contracting, Neoliberalism, Political parties

## Abstract

**Background:**

This study analyzed several political determinants of increased private-sector management in Brazilian health care. In Brazil, the poor depend almost exclusively on the public Unified Health System (the SUS), which remains severely underfunded. Given the overhead costs associated with privately contracted health services, increased private management is one driver of higher expenditures in the system. Although left parties campaign most vocally in support of greater public control of the SUS, the extent to which their stated positions translate into health care policy remains untested.

**Methods:**

Drawing on multiple publicly available data sources, we used linear regression to analyze how political party-in-power and existing private sector health care contracting affect the share of privately managed health care services and outsourcing in municipalities. Data from two election periods—2004 to 2008 and 2008 to 2012—were analyzed.

**Results:**

Our findings showed that although private sector contracting varies greatly across municipalities, this variation is not systematically associated with political party in power. This suggests that electoral politics plays a relatively minor role in municipal-level health care administration. Existing levels of private sector management appear to have a greater effect on the public-private makeup of the Brazilian healthcare system, suggesting a strong role of path dependence in the evolution of Brazilian health care delivery.

**Conclusion:**

Despite campaign rhetoric asserting distinct positions on privatization in the SUS, factors other than political party in power have a greater effect on private-sector health system management at the municipal-level in Brazil. Given the limited effect of elections on this issue, strengthening participatory bodies such as municipal health councils may better enfranchise citizens in the fundamental debate over public and private roles in the health care sector.

## Background

In Brazil, grievances about mismanagement and lack of quality in health care provision exploded in mass protests in 2013. A vocal left argued that the proliferation of private sector stakeholders alongside public sector provision was driving up overhead costs and complicating the structure of Brazilian healthcare delivery. However, the role of elected officials in driving or slowing this phenomenon is unclear.

This study examined whether and how political party in power influences public-private contracts in health care delivery in Brazil. We compared whether municipal election results, or existing health system characteristics, played a greater role in determining the extent of private sector involvement in health care provision. While previous literature has examined local determinants of health system composition, and the overall distribution of health care contracts in Brazil, no existing studies, to our knowledge, analyze the effect of partisanship on health care privatization in Latin America’s largest country and biggest economy [1–7]. Brazil’s health system is a decentralized, two- part public-private system that grants access to care to the poor and the wealthy through two parallel yet partially overlapping tracks. It does this through a network of public facilities that provide access to care to all Brazilians free of cost, and a parallel private system of operators that limit access to care primarily to those who hold supplementary private health plans or can afford to pay out of pocket. There is some overlap, as individuals with private plans are also entitled to use public facilities, and public-only patients may use a limited number of providers that contract with the government system. Against this backdrop, greater evidence is needed on the political determinants of decisions that may shift or further entrench this divided system. To what extent do electoral politics play a role in strengthening or weakening private health care management in Brazil? This study provides evidence on whether elections truly matter for private-sector health care contracting in Brazil.

### Challenges facing the Brazilian health system

The Unified Health System (Sistema Único de Saúde, or SUS) is simultaneously a source of pride and concern for Brazilians. The SUS boasts a wide network of clinics, health posts, and programs that grant universal access to an array of care free of cost, at the point of service. Despite the broad accessibility of the SUS, widespread reports of poor quality, government underinvestment and mismanagement of funds persist [8]. A majority of Brazilians (77%) are dissatisfied with the quality of hospital care in the country [9].

Alongside a struggling public system, private ownership and management has increased in health care facilities and in the health care equipment and technology sector [10]. Between 2006 and 2012, the total quantity of privately managed and maintained equipment in the SUS nationwide increased by approximately 88%, while publicly managed equipment grew by only about 51% [11]. In the decentralized configuration of the SUS, municipal governments have substantial latitude in financial and administrative decisions, and can decide to initiate and maintain contracts with the private sector in health care delivery and administration [5, 12, 13].

At every level of government, political parties and civic groups voice opposition to the expansion of private health care management. These include left parties such as the Socialism and Freedom Party (PSOL) and until recently, the majority of the Worker’s Party (PT), as well as civil society organizations such as the *Frente Nacional Contra a Privatização da Saúde* (National Front Against the Privatization of Health). These groups contend that increased private-sector contracting privileges a few stakeholders and distorts the intended public nature of the SUS, on which poorer Brazilians are dependent. Yet, it remains unclear whether political parties have staked out distinct positions on the public-private composition of the SUS. Further, it is unclear whether administrative choices at the local level reflect party ideology, or respond to local supply and demand factors.

### History of private sector participation in the SUS

The extant public-private composition of Brazilian health system is rooted in the political history of the country. The “*sanitarista”* movement—a national political movement driven by public health professionals—pushed to include an ambitious universal health care provision in the 1988 Constitution. As a result, Article 196 of the Constitution defines access to health care as a right that the state must provide. The government created the SUS in 1990 to carry out this duty [10].

The current configuration of the SUS was also shaped by the health care system that preceded it. The SUS replaced the health insurance scheme established in the 1960s by the military dictatorship, which financed individual health care provision through the social security system. This system, the INAMPS (Instituto Nacional de Assistência Médica da Previdência Social), only covered workers employed in the formal sector. Under INAMPS, Brazil witnessed the proliferation of private hospitals, which contracted with the government [14]. This was in part due to a policy in the 1970s that subsidized their construction. By 1989, private hospitals accounted for about 80% of all hospitals [15, 16].

Over the last two decades, a growing number of middle- and upper class Brazilians have purchased private insurance, either individually or through their employers. Between 2003 and 2012, the number of Brazilians holding private plans increased 53%, from 32 to 49 million [17]. As a result, over 25% of the Brazilian population also has access to exclusively private sector health care services paid for by a private insurer. Moreover, the Brazilian government also offers a federal tax incentive exempting all private out of pocket health care expenses from taxation. This tax subsidy serves, in practice, to promote private health expenditures and to shift higher income earners, who stand to gain most from the tax break, from the public to the private sector. As a result, the increase in access to private sector care is a middle- to upper- middle class phenomenon. The poor in Brazil continue to depend almost exclusively on public facilities in the SUS for care.^1^

In addition to the private health care that is exclusive to holders of private plans, an array of private companies are involved along different points of the delivery of publicly financed care. Privately managed health care facilities and equipment also play a growing role in publicly-funded care*.* Between 2006 and 2010, the number of public outpatient facilities in Brazil plateaued, while private facilities continued to grow [10]. The faster growth of private facilities is noteworthy because although the majority of SUS-financed inpatient care takes place in private hospitals, most private, for-profit outpatient clinics are for exclusive use of private plan holders [12]. Due to geographic socioeconomic inequalities in Brazil, the need for public facilities is greater in rural areas and in the Northeast region, where the proportion of individuals holding private plans is much lower [18].

**Fig. 1:**
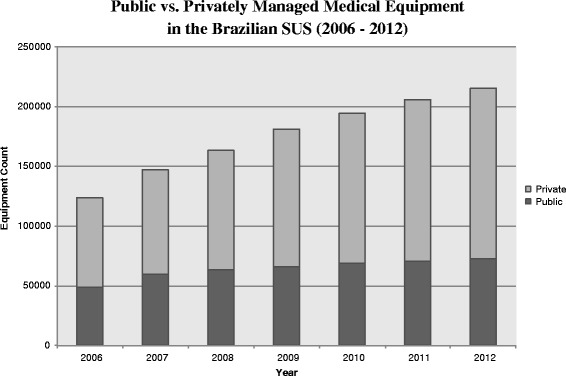
Composition of public and private medical equipment (of all types) in the Brazilian SUS between 2006 and 2012. Cadastro Nacional de Estabelecimentos de Saúde (CNES), Brazilian Ministry of Health

A similar trend in private ownership and management took place with medical equipment, ranging from mammogram screening tools to dental equipment [19]. The coexistence of public and private ownership and management of medical equipment in Brazilian health care delivery is widespread (Figure 1). However, the drivers of the growth and local level variation in government procurement of privately managed health care services is less understood.^2^

### Politics of the privatization debate

The viewpoint that nations should shift the provision of publicly-provided services to the private sector is central to the economic philosophy of neoliberalism [20, 21]. Neoliberal reforms became commonplace in Latin American countries in the 1980s under the influence of the World Bank and the International Monetary Fund (IMF) [22–24]. As a proposed solution to high levels of national debt and a dire need for infrastructure, multilateral lenders following the Washington Consensus believed that relegating public services to private companies, which vie for government contracts, would introduce greater competition, efficiency and quality in public service provision [4, 22, 24–27]. The role of IMF and World Bank structural adjustment programs in economic development and fiscal stabilization remains contentious and,whether politicians align themselves with a neoliberal agenda, or support a strong role of the state is politicized in many domestic policy domains. In Brazil, where public participation in the allocation of government resources was a key demand of the social movements that created the SUS, the debate over whether health policy reforms are too strongly influenced by outside actors and neoliberal ideology polarizes the left and right.

Brazil stands out among Latin American countries for adopting a rights-based public health system at the end of the military dictatorship in 1988, but debates continue over whether political leaders prefer privatization reforms in everyday health care management and delivery. The political left voices a fierce desire to guard the public nature of the system, and proponents lean on the Brazilian Constitution for support [28]. Despite the historically entrenched network of private hospitals left over from the military regime, the 1988 Constitution contains provisions to guarantee a health care system that is largely public in nature. Article 196 of the Brazilian Constitution asserts that the provision of health care is the duty of the state, and Article 199 specifies that for-profit, private sector companies should not receive public funds from the SUS [10, 29]. Further, Article 199 declares that the private sector should remain “complementary” to the SUS. However, the issue has become more nuanced in recent years, with the debate on private management contracts (or *contratos de gestão)* within the SUS [30].

The reliance on private sector entities—both for-profit and non-profit—to carry out administrative and technological functions within the health system is a point of disagreement among actors on the left. Although liberal supporters of private sector contracting often argue that involving the private sector can increase the quality and efficiency of health delivery, this view faces several counterarguments [31]. Arguments against private contracting contend that contracts make the SUS more fragmented, with many competing points-of-view operating in the same system, and that it decreases public control of the SUS’s public funds. Private sector health spending has also been shown to extract greater expenditures due to increased overhead costs and a tendency toward overutilization [32]. Though left parties in Brazil campaign against neoliberal policy positions, it is unlcear whether they distinctly avoid contracting with private companies in health care provision.

### Political parties in Brazil

There are more than 30 political parties registered in Brazil’s multiparty, federalist system, and parties frequently govern in coalition [33]. However, literature is inconclusive on the extent to which party discipline and cohesive policy behavior are evident among politicians belonging to the same political party. One strand of the literature contends that clientelism—the distribution of goods in exchange for electoral support—drives the behavior of Brazilian political elites and undermines the strength of political parties [34–36]. This strand argues that parties lack the distinctions in ideology or party discipline to send coherent messages on policy positions to the electorate [37–39].

A competing view argues that political parties have become stronger over the years [40]. By this argument, politicians invest in “creating value for the party brands” as a means of distinguishing themselves programmatically from other parties [40]. This party-oriented shift became more viable after larger scale policy reforms rendered small-scale forms of patronage less valuable for politicians. For example, the long-term electoral rewards that parties receive from providing a widely successful social services—“Bolsa Família” (Family Income Support) for example— is more valuable to the party brand than small acts of patronage. This paper leans on the latter strand of the literature and takes the hypothesis that there is an association between poltical party and health policy decisions.

Although disagreement exists on whether Brazilian parties behave ideologically, Brazilian parties self-identify along a left-right ideological spectrum [3, 37, 41–43]. Because of the uncertainty around party adherence to ideological placements, we do not categorize parties based strictly on ideology in the statistical model. However, understanding where political parties lie on this spectrum is important for contextualizing study findings.

## Methods

We approached the study design with two competing hypotheses in mind. First, we hypothesized that left-of-center political parties will demonstrate greater support for public sector health services, and thus be associated with fewer contracted private sector health care services. We based this hypothesis on recent theories that contend that Brazilian political parties increasingly demonstrate greater cohesion and programmatic behavior [2, 40]. The alternative hypothesis held that baseline institutional arrangements—the existing level of public-private health care contracts—would be a more important determinant of public-private health care management contracts at the end of a mayoral administration. The second hypothesis relied on theories of path dependence. Although municipal administrations have the power to reexamine and change contracts annually, existing policy arrangements, and the powerful interests that arise from them, may “lock-in” a status quo from which it is politically difficult to depart [44, 45].

To test these hypotheses, we analyzed the public-private composition of healthcare services at the end of mayoral administrations. We aimed to estimate whether ideologically distinct political parties behave differently in the domain of private sector contracting in health care, and if not, which factors appear to drive differences in private health care administration in Brazil.

### Model building process

This study used linear regression to analyze the effect of political party in power and other municipal-level determinants on the level of private sector involvement in the Brazilian SUS. Using data from the 2004 and 2008 election cycles, we constructed models to predict the share of private management of health equipment, health care outsourcing and health spending that exists at the end of a mayoral term.

### Measures of private-sector healthcare administration

We chose five dependent variables to measure the degree of private sector contracting and administration in health care. First, we selected three widely used types of medical equipment—dental equipment, ultrasounds, and x-rays—and identified the proportion of these equipment that were privately owned, managed and maintained. The majority of Brazilian municipal health systems have ultrasounds, x-rays, and dental equipment whereas less equitably distributed medical equipment (such as Magnetic Resonance Imaging machines or MRIs) do not exist in resource poor municipalities.^3^ Overall, very profitable equipment types (such as imaging services) that are densely concentrated in a few wealthy municipalities, tend to be reimbursed above cost by the SUS, and would likely be particularly sensitive to private sector contracts regardless of existing local supply [46].

The percentage of private hospital beds, out of total hospital beds, is included as a measure of private sector dominance of hospital care. We obtained data on private medical equipment and hospital beds from the *Cadastro Nacional de Estabelecimentos de Saúde* (CNES), or National Registry of Health Establishments, maintained by the Brazilian Ministry of Health [11].

The fifth outcome variable measured the proportion of health care expenditures allocated to outsourced service corporations. We calculated this value for all municipalities in the baseline years 2004 and 2008 and the end of mayoral terms, in 2008 and 2012. We used data from the *Sistema de Informações Sobre Orçamentos Públicos em Saúde* (SIOPS), or Information System on Public Health Budgets database, provided by the Ministry of Health [47].

Finally, we included a sixth model to estimate health care expenditures per capita to compare whether political party support for privatization and for health care expenditures overall. To maintain comparability of outcomes, we expressed health care expenditures as a percentage of average health expenditures, across municipalities.

### Key explanatory variables

**Fig. 2:**
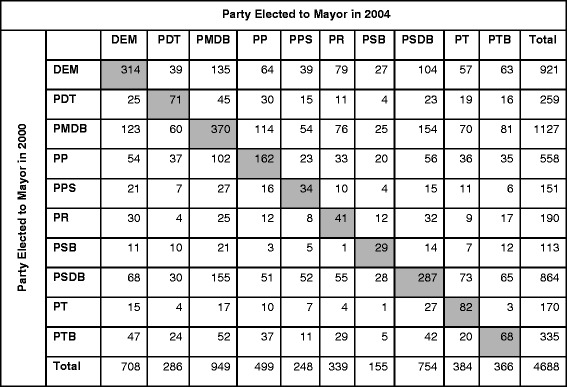
Summary of Party Transitions for Mayoral Seats from 2000 to 2004. Only the top 10 political parties represented in Brazilian municipalities are included. Tribunal Superior Eleitoral (TSE)

**Fig. 3:**
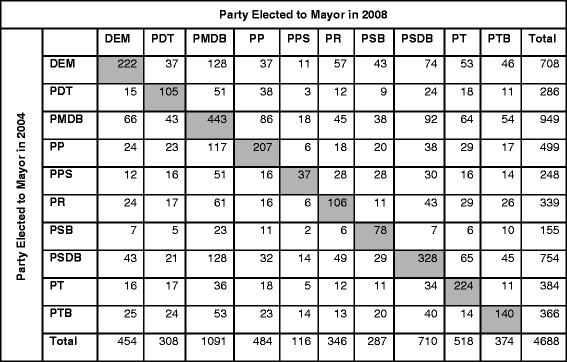
Summary of Party Transitions for Mayoral Seats from 2004 to 2008. Only the top 10 political parties represented in Brazilian municipalities are included. Tribunal Superior Eleitoral (TSE)

Our primary key predictor is *Political Party in Power,* which is defined as the party of the mayor elected in 2004 and 2008. The mayor’s party plays an important role in choosing the municipality’s Secretary of Health (either of the mayor’s party or of a coalition party), who oversees the administration and management of the local health care system. Our analysis included the top ten political parties as determined by nationwide representation. Limiting the analysis to 10 of the over 30 Brazilian parties left a substantial sample, as these parties were elected to mayor in over 4600 (close to 85% of) municipalities in 2004, governing about 83% of the national population. Their reach in municipal power grew in 2008, as mayors from these parties were elected in 5176 municipalities, accounting for approximately 93% of the Brazilian population (using 2010 population numbers) [33]. This growth in municipal power was, in part, due to the merger of two conservative political parties (Party of the Reconstruction of the National Order, or PRONA and the Liberal Party, or PL) into the Party of the Republic (PR) party, as well as the increased election of the PT and Brazilian Labor Party (PTB) at the municipal level. As shown in Figs. 2 and 3, incumbent parties held onto a sizable proportion of mayoral seats across elections (31% in 2004 and 40% in 2008). To account for the potential effect of incumbency on local health care privatization, we included a covariate for *Incumbency* in the model. We retrieved elections data from the Tribunal Superior Eleitoral (TSE), the Supreme Electoral Court, which maintains a public database on local and national elections in Brazil [48].

As a secondary key predictor, we included *Baseline Level of Private-Sector Involvement* for each of five response variables to account for the public-private contracting arrangement that existed before each election period. We included the baseline (2004 and 2008) measures for the three equipment types, hospital beds and outsourcing, to test our second hypothesis, that the existing saturation of private sector healthcare contracting reflects a stakeholder environment where the continuation of such agreements is politically difficult to disrupt.

### Covariates

We included additional covariates to analyze the effect of the existing supply of health care equipment and other factors that may generate a demand for private care in a municipality. For models estimating privatization in medical equipment and hospital beds, we included measures for total existing supply (per capita) of the given type of equipment. We incorporated this measure to capture any existing deficit in health care equipment that may have existed at baseline in a municipality. A deficit in health equipment could create a demand for an urgent solution from government, which may include contracting the technology and its maintenance from the private sector.

The model also controlled for the Human Development Index (HDI) to account for the fact that more advanced specialist and tertiary care options are in greater supply in more developed, urbanized areas [3]. Last, we controlled for salary for the top of 4th quintile of income earners in each municipality. This variable provides a measure of the economic standing of the upper middle-class individuals by municipality, and is a proxy for the proportion of municipal residents who hold private health plans. The proportion of private health plans could also drive the level of privately managed and provided health care services that are available.

Using this general model, we conducted linear regression analyses for each of the six outcome variables of interest. We input values at the end of each mayoral term for response variables, and for explanatory variables we used baseline measures from election years. For socio-demographic data, such as population, HDI, and upper middle class income, we used the most recently released data from the 2010 Brazilian Census. Because the Brazilian census is decennial, we used sociodemographic data from 2010, which is the nearest year to the mayoral terms we analyzed. We deleted cases based on availability of data.

## Results

### Descriptive statistics

In aggregate, there was an increase in privately managed equipment of all types between 2006 and 2012 in Brazil (Figure 1). Of all the domains studied, the relative share of privately managed dental equipment increased most substantially (by more than 5%) over that time period (Table 1). Two of the five dimensions—ultrasounds and hospital beds—showed an overall decrease in average percentage of municipal-level private sector management across the two periods. The decrease in percent private hospital beds was expected given that there has been a steady increase in public beds since the advent of the SUS [10]. The reduction in the number of private hospitals contracting with the SUS made an increased proportion of public beds possible [10]. Table 1 suggests that contracting of private dental equipment may be driving the aggregate increase in privately managed equipment in the SUS.^4^

**Table 1:** Average %Private Sector Involvement in Health Care and Health Care Spending by Year ^a,b,c^

Year	Equipment				Outsourcing (% health expenditures)	Health Spending per capita (2012 $R)
	Dental (% Private)	X-Rays (% Private)	Ultrasounds (% Private)	Hosp. Beds (% Private)		
2008	12.3	34.8	35.8	21.9	14.5	312.4
	(19.4)	(68.4)	(72.7)	(36.6)	(12.9)	(155.4)
2012	17.5	35.4	34.7	18.4	16.4	540.0
	(33.3)	(69.7)	(71.4)	(21.6)	(15.2)	(260.3)
Change	5.2	0.6	−1.1	−3.5	1.9	+227.6

^a^Data are presented as means (interquartile range)

^b^% Private denotes percent privately managed and maintained

^c^Data Source: National Registry of Health Establishments and Information System on Public Health Budgets, Ministério da Saúde, Brasil

For some domains, overall private sector management changed only slightly over the two election periods, but the variation across municipalities was remarkable. For example, privately managed x-rays and ultrasounds remained at about 35% for both election periods, and changed by only 0.6% and −1.1% respectively. However, the interquartile ranges for private management of both types of equipment were quite large at 69.7% and 71.4%, respectively. This reflects that in some municipalities, equipment types are entirely privately managed, and in others they are completely public. The regression analysis that follows sought to explain some of the determinants of this variation.

### Regression analysis

Overall, we found that political party-in-power did not have a consistent effect on the proportion of privately managed health care services in a municipality. This is contrary to our hypothesis that Brazilian parties behave programmatically in policy making, but it supports earlier theories on the lack of ideologically consistent policy positions by Brazilian political parties. However, regression analyses upheld our second hypothesis that existing public-private arrangements were more strongly associated with the level of private sector health care contracting at the end of a mayor’s administration. Tables 2, 3, 4 and 5 depict the results of a series of linear regression analyses for both election periods.

**Table 2:** Party-in-power estimates of linear regressions predicting 2012 level of privatization and health care expenditures by party elected in 2008 (controlling for incumbency, baseline privatization, HDI, and upper middle-class income)^a^

Party Elected In 2008	Dental	X-rays	Ultrasounds	Hospital Beds	Outsourcing	Health Expenditures
DEM	−0.86 (0.55)	−0.40 (1.02)	1.12 (1.27)	−0.11 (1.16)	−0.35 (0.46)	−.69 (1.19)
PDT	0.15 (0.66)	0.40 (1.21)	1.43 (1.50)	−0.20 (1.33)	−0.23 (0.54)	−2.84* (1.40)
PMDB	−0.02 (0.39)	−0.30 (0.71)	0.32 (0.88)	0.50 (0.79)	−0.45 (0.33)	−0.07 (0.84)
PP	0.21 (0.54)	0.34 (0.97)	−0.81 (1.24)	−1.57 (1.10)	−1.00* (0.45)	−0.48 (1.16)
PPS	−0.16 (1.03)	1.51 (1.84)	−0.72 (2.27)	1.60 (2.10)	0.58 (.84)	−0.29 (2.20)
PR	0.26 (0.62)	−1.28 (1.11)	−2.45^.^ (1.38)	0.90 (1.23)	0.06 (0.52)	1.73 (1.35)
PSB	0.024 (0.68)	0.80 (1.31)	0.49 (1.45)	0.02 (1.30)	0.55^·^ (0.57)	1.05 (1.47)
PSDB	−0.25 (0.46)	−1.67* (0.81)	−2.12* (1.03)	−0.67 (0.96)	0.21 (0.38)	2.02* (0.99)
PT	0.40 (0.52)	0.09 (0.92)	3.51** (1.14)	0.32 (1.01)	1.21** (0.44)	0.11 (1.13)
PTB	0.24 (0.60)	0.51 (1.17)	−0.77 (1.40)	−0.78 (1.25)	−0.58 (0.51)	−0.52 (1.30)
F statistic	40.28	71.60	209.84*	43.86	191.80*	114.11
Num. obs.	4650	3075	2429	2987	4347	4476

^a***^ *p* < 0.001, ^**^ *p* < 0.01, ^*^ *p* < 0.05, ^·^ *p* < 0.1. Entries are deviations from the mean (standard errors) times 100

**Table 3:** Covariates in linear regression models predicting 2012 Levels of Privatization by Party in Power in Brazilian Municipalities^a^

	Dental	X-rays	Ultrasounds	Hospital Beds	Outsourcing	Health Expenditures
Incumbency	0.17	−0.07	0.43	−1.90^*^	−0.46	0.75
	(0.37)	(0.66)	(0.82)	(0.74)	(0.30)	(0.83)
HDI	0.50^***^	0.44^***^	0.27^*^	0.11	0.21^***^	0.31^*^
	(0.06)	(0.11)	(0.13)	(0.12)	(0.05)	(0.10)
Upper middle class income	0.05^***^	0.04^**^	0.07^**^	0.05^·^	−0.01	0.08^**^
	(0.01)	(0.02)	(0.03)	(0.03)	(0.01)	(0.03)
Controls for Baseline and Aggregate Change^b^						
Total Equipment per capita end of term	2.40^***^	4.29^***^	3.45^***^	0.35^***^		
	(0.08)	(0.28)	(0.50)	(0.05)		
Total Equipment per capita baseline	−2.91^***^	−4.32^***^	−3.38^***^	−0.30^***^		
	(0.09)	(0.29)	(0.43)	(0.04)		
% Private Equipment baseline	0.83^***^	0.79^***^	0.77^***^	0.75^***^		
Interquartile Range	0.19	0.68	0.72	0.37		
	(0.01)	(0.01)	(0.01)	(0.01)		
Outsourcing/Health Expenditures					0.63^***^	
					(0.01)	
Health Expenditures per capita						0.77^***^
						(0.01)
R^2^ Full Model	0.76	0.76	0.73	0.67	0.44	0.69
R^2^ Political Variables Only	0.01	0.01	0.02	0.01	0.01	0.01
Num. obs.	4650	3075	2429	2987	4347	4476

^a***^ *p* < 0.001, ^**^ *p* < 0.01, ^*^ *p* < 0.05, ^·^ *p* < 0.1. Entries are regression coefficients (standard errors). Note: Incumbency estimates multiplied by 100

^b^Estimates for total equipment per capita at end of term and baseline should be considered together. When combined, their sum equals the effect size for the change in the total stock of equipment over the mayoral term

**Table 4:** Difference in 2008 % privatization and health care expenditures by party elected in 2004 (controlling for incumbency, baseline privatization, HDI, and upper middle-class income)^a,b^

	Dental	X-rays	Ultrasounds	Hospital Beds	Outsourcing	Health Expenditures
Party Elected In 2004						
DEM	−0.78	1.81^.^	2.45^.^	−0.35	−0.41	−0.94
	(0.54)	(1.07)	(1.26)	(0.96)	(0.36)	(1.18)
PDT	−0.17	−0.59	−0.72	0.87	1.38**	2.04
	(0.69)	(1.43)	(1.82)	(1.34)	(0.53)	(1.72)
PMDB	−1.31**	−0.13	0.06	1.21	0.02	0.57
	(0.44)	(0.90)	(1.17)	(0.83)	(0.32)	(1.04)
PP	−0.86	−2.07^.^	−2.40	−3.14**	−0.38^.^	1.89
	(0.56)	(1.16)	(1.48)	(1.09)	(0.41)	(1.34)
PPS	−0.24	−0.39	−1.03	−1.62	1.06	−1.48
	(0.75)	(1.49)	(1.84)	(1.37)	(.56)	(1.84)
PR	0.12	1.99	0.75	1.61	−0.64	−1.36
	(0.71)	(1.42)	(1.69)	(1.28)	(0.49)	(1.60)
PSB	1.06	0.50	1.90	2.08	−0.21^·^	−0.54
	(1.19)	(2.31)	(2.52)	(1.73)	(0.70)	(2.31)
PSDB	0.50	−1.12	−1.22	0.53	−0.27	−1.42
	(0.48)	(1.00)	(1.18)	(0.91)	(0.35)	(1.13)
PT	1.00^.^	2.01	−0.13	0.16	0.39	−1.71
	(0.61)	(1.23)	(1.49)	(1.15)	(0.46)	(1.52)
PTB	0.68	−1.93	0.48	−1.34	−0.93*	2.94^.^
	(0.66)	(1.36)	(1.65)	(1.28)	(0.47)	(1.54)
F statistic	201.65*	151.47	93.81	176.11^.^	187.60^.^	125.74
Num. obs.	3420	2840	1988	2998	4480	4534

^a***^ *p* < 0.001, ^**^ *p* < 0.01, ^*^ *p* < 0.05, ^·^ *p* < 0.1. Entries are deviations from the mean (standard errors) times 100

^b^Due to limitations in data availability, 2005 baseline values were used for x-rays, ultrasounds, and hospital beds, 2006 values for dental equipment, and 2004 values for outsourcing and health expenditures

**Table 5:** Results of linear regression models predicting 2008 Levels of Privatization by Party in Power in Brazilian Municipalities^a^

	Dental	X-rays	Ultrasounds	Hospital Beds	Outsourcing	Health Expenditures
Incumbency	−0.27	−0.35	0.85	−0.85	−0.46	0.02^.^
	(0.42)	(0.86)	(1.10)	(0.01)	(0.00)	(0.01)
HDI	0.19^**^	0.23^.^	0.62^***^	−0.07	0.16^***^	0.79^***^
	(0.06)	(0.13)	(0.15)	(0.12)	(0.04)	(0.14)
Upper middle class income	0.09^***^	0.08^**^	0.06^.^	0.06^*^	0.04^***^	−0.16^***^
	(0.01)	(0.03)	(0.04)	(0.03)	(0.01)	(0.04)
Controls for Baseline and Aggregate Change^c^						
Total Equipment per capita end of term	0.92^***^	4.89^***^	8.85^***^	0.13^***^		
	(0.11)	(0.30)	(0.87)	(0.03)		
Total Equipment per capita baseline	−0.78^***^	−4.73^***^	−10.37^***^	−0.07^*^		
	(0.11)	(0.29)	(0.85)	(0.03)		
% Private Equipment	0.78^***^	0.77^***^	0.72^***^	0.80^***^		
Interquartile Range	0.20	0.67	0.73	0.47		
	(0.01)	(0.01)	(0.01)	(0.01)		
Outsourcing/Health Expenditures					0.44^***^	
					(0.01)	
Health Expenditures per capita						0.70^***^
						(0.01)
R^2^ Full model	0.77	0.70	0.70	0.71	0.47	0.60
R^2^ Political variables only	0.02	0.01	0.01	0.01	0.03	0.01
Num. obs.	3420	2840	1988	2998	4480	4534

^a***^ *p* < 0.001, ^**^ *p* < 0.01, ^*^ *p* < 0.05, ^·^ *p* < 0.1. Entries are regression coefficients (standard errors). Note: Incumbency estimates are multiplied by 100

^b^Due to limitations in data availability, 2005 baseline values were used for x-rays, ultrasounds, and hospital beds, 2006 values for dental equipment, and 2004 values for outsourcing and health expenditures

^c^Estimates for total equipment per capita at end of term and baseline should be considered together. When combined, their sum equals the effect size for the change in the total stock of equipment over the mayoral term

We found very few significant relationships between political party in office and private sector involvement in health care. However, the most significant findings for parties entering in 2008 were among the Party of Brazilian Social Democracy (PSDB) and the Worker’s Party (PT). As shown in Table 2, PSDB in power was significantly associated with fewer privately managed medical equipment (1.7% fewer x-rays and 2.1% fewer ultrasounds than the average across all political parties). Interestingly, the association between the PT—the left party with the most political power in Brazilian government—and health care privatization was greater than other parties with respect to ultrasounds and outsourcing. The association with the PT and ultrasounds has a relatively large deviation (+3.5%) from the average in the direction of more privatization. The association between the PT and health care outsourcing is 1.21% greater than the average across all parties studied. These findings are somewhat striking, as the PSDB is generally known to be more supportive of neoliberal policies, while the PT postures as a staunch defender of the public sector in health care [49].^5^

The effect of party elected in 2004 on 2008 levels of private sector involvement in health care is even less clear (Table 4). No single party is associated with significant privatization outcomes on more than one domain. The conservative Democratas (DEM) party comes closest to an association across multiple domains with higher than average levels of privatization in both x-rays (2%) and ultrasounds (2.6%). However, for both outcomes, this association is only significant at the *p* < .10 level. With other strong associations found on only single dimensions, it is difficult to make an interpretation of a party demonstrating a stance on health care privatization during this term.

Incumbency—which is widespread in Brazilian municipal elections (Figs. 2 and 3)—did not have a significant effect on most of the health policy outcomes. However, for private hospital beds (especially in the 2008 election), incumbency does have a strong and statistically significant effect. The strong negative association between incumbency and proportion of private hospital beds suggests that political administrations may offer greater public access to hospital facilities as a means to hold onto office. Alternatively, it could suggest that incumbents hold onto office because they offered greater access to hospital facilities for those who lack access to private care.

Overall, municipal level development (HDI), socioeconomic characteristics, and baseline private sector involvement explained most of the variation seen in private sector health care management and health expenditures. Baseline levels of private sector involvement, existing health equipment per capita, and HDI were the strongest predictors of level of health care privatization (Tables 3 and 5). Moreover, R-squared values for predictor-only models including party of the mayor elected and incumbency dramatically increased (from 0.01 to 0.77 in the case of Dental Equipment) with the addition of covariates. This confirmed that, relative to the effects of local supply and demand factors, political party in power explained very little of the mix of private sector health care contracting across Brazilian municipalities.

Consistently, higher *Baseline Level of Private Sector Involvement* was positively and significantly associated with private sector management for dental equipment, x-rays, ultrasounds, and hospitals beds at the end of the electoral term. The 0.83 parameter estimate for this variable in the dental equipment model, means that a difference in privately managed dental equipment of 10% in 2008 is associated with an 8.3% greater proportion of privately management dental equipment in 2012 (Table 3). Estimates of percent privately managed equipment for the four equipment outcomes ranges from .75 to .83. Thus a strong relationship exists between high levels of existing private sector contracting and greater levels of future private sector health contracts. Although this model cannot prove direction of causality, these findings suggest a strong role of path dependence. In other words, constraints of existing institutional arrangements on the set of future possible arrangements determines which municipalities opt for privately managed health care services versus publicly managed.

Also significant is that existing levels of total equipment, at baseline, is consistently negatively associated with private management levels for all types of medical equipment. This association suggests that municipalities that started with lower levels of equipment per capita have a stronger association with private-sector contracting. Thus, a deficit of health care equipment may put pressure on municipalities to seek privately managed equipment to fill the gap. HDI and upper middle class income have slight, but, statistically significant effects on greater percent private sector management for most of the response variables. The positive direction of this relationship is consistent with the logic that greater levels of development and urbanization in a municipality and higher income levels are associated with a greater demand for health care services, both public and private.

## Discussion

It is not entirely surprising that Brazilian parties show ideologically inconsistent behavior in health system funding and administration in the current policy context in Brazil. First, the relative low magnitude of change across election years also suggests that existing federal policies constrain the latitude that any given mayoral administration or city council has to significantly change municipal budgets. Embedded in the design of the decentralized SUS is a formula based system of financing that renders many municipalities financially dependent on federal- and state- level transfers in order to finance and run their local health systems [1]. These financial transfers are based on population and revenue. Therefore, poorer municipalities have less flexibility in whether or not to contract with private health equipment providers. Municipalities are also constrained in their budgetary expenditures by the 2000 *Lei de Responsibilidade Fiscal* (Law of Fiscal Responsibility). This law does not allow municipalities to run deficits. Additionally, there are specific requirements for budget allocations to local activities. Thus, the finding that a municipality’s material conditions—such as HDI and existing health equipment stock—are stronger predictors of percent privately managed health services likely reflects that economic and regulatory constraints considerably limit the autonomy of any mayoral administration to alter the existing public-private composition of the health system. Under these circumstances, even an administration that wanted to move away from the private sector may lack feasible alternatives.

Second, the lack of distinct health care contracting associations by political party is in line with the literature describing the predominance of clientelism, or patronage-driven political behavior, in Brazilian politics. This would appear to explain why the existing health-care contracting environment is the single largest and most significant association between contracts at the beginning and end of a given administration. It is particularly striking that this does not only hold when incumbents hold onto power, but even when party-in-power changes. Given that contracts are up for reconsideration and renewal annually, this finding suggests a considerable degree of stickiness of the status quo of Brazilian health care contracting in municipalities.

In addition, the contractual landscape that dominates a municipality can generate powerful incentives to maintain the status quo, even for leftist politicians. For example, the association of the PT with greater privatization suggests that political parties who claim to be further to the left but who govern in relatively wealthy municipalities may concede to the pragmatism of contracting with private providers. Although we cannot claim causality, this finding overlaps with accounts that the PT, for example, has moderated its positions over time, departing from its radical, grassroots past [50]. The high demand for medical devices in Brazilian health care is key in this story. With the increasing role of medical technology in health care, and the growing prevalence of chronic diseases in Brazil’s population of over 200 million, local governments must meet these demands by drawing on public and private sector resources [10, 46]. Although mayors have the power to cancel contracts during annual reevaluations, this often does not occur due to lack of other alternatives. The gap between supply and demand in services like imaging, dental care and others certainly lends itself to the political pragmatism suggested by our study results.

No major Brazilian political party exhibits stubborn defiance to granting the private sector a growing role in the SUS or keeping it complementary as Article 199 of the Brazilian constitution outlines [51]. Perhaps one reason for this is that contracting is not seen as interfering with access, so long as SUS patients are able to access private technologies. The one indicator of access to the system by the poor (SUS only patients) that we analyze, hospital beds, appears to be moving in the direction of greater public access.

### Study limitations

One limitation of this study lies in its inability to estimate the effect of more than one political party in power, given that multi-party coalitions govern a substantial percentage of Brazilian municipalities. Ideally, we would be able to gauge the party affiliation of the Secretary of Health, who is chosen by a member of the ruling party. Such a measure might reflect an average ideological score across the members of the governing party, but this average ideology may not better approximate which party chooses the secretary of health. Overall, the Executive retains considerable policymaking power in Brazilian cities, particularly through the Secretariat of Health. Therefore, using mayor’s party as an indicator of the parties-in-power remains a logical choice for the political variable.

We are also limited by the absence of data on the role of municipal level health councils. Health councils serve as a conduit for civil society involvement in the health policy process as specified in the 1990 Health Statute (or *Lei Orgânica da Saúde*). Given the limited choice that citizens appear to have at the ballot box to vote on their preferences for private sector involvement in the SUS, these vehicles for participatory policy making seem all the more crucial for local health care policy making. However, detailed data on the positions of these councils was not available for this study [52].

## Conclusions

In Brazil, where left-leaning politicians make frequent claims to preserve the public nature and accessibility of the health care system, municipal governments were not associated with significant partisan behavior in private sector contracting in health care between 2004 and 2012. Despite political campaign rhetoric, existing institutional arrangements at the national and local level appeared to drive future municipal level public-private contracts. As the level of private sector contracting in health care increases, party ideology appears less important than other factors in determining whether local health system equipment needs are addressed through the private sector. Under rules set by the government itself, the private sector seems poised to eclipse the public sector in some domains of health care delivery. National policy constraints and the entrenchment of public-private contracting suggests that, without a radical departure from current practices, we should expect privatization within the SUS to continue well into the future. In the Brazilian SUS, where social participation in the policy process is of paramount importance, strengthening bodies such as municipal health councils may better enfranchise citizens in the fundamental debate over public and private roles in the health care sector.

## Abbreviations

CNES: *Cadastro Nacional de Estabelecimentos de Saúde* National Registry of Health Establishments
DEM: Democrats Party
HDI: Human Development Index
HMOs: Health Mainenance Organizations
INAMPS: Instituto Nacional de Assistência Médica da Previdência Social, National Institute of Medical Social Security Assistance
MRI: Magnetic Resonance Imaging machine
PDT: Democratic Labor Party
PMDB: Party of the Brazilian Democratic Movement
PP: Progressive Party
PPS: Popular Socialist Party
PR: Party of the Republic
PRB: Brazilian Republican Party
PSB: Brazilian Socialist Party
PSDB: Party of Brazilian Social Democracy
PSOL: Socialism and Freedom Party
PT: Worker’s Party
PTB: Brazilian Labor Party
SIOPS: *Sistema de Informações Sobre Orçamentos Públicos em Saúde*, or Information System on Public Health Budgets
SUS: *Sistema Único de Saúde,* Unified Health System
TSE: Tribunal Superior Eleitoral, the Supreme Electoral Court

## Footnotes

1 According to a 2012 analysis by the Center of Public Policies of Insper (Instituto de Ensino e Pesquisa) entitled “O Panorama da Saúde do Brasil,” (The Health Panorama in Brazil) 87% of the poorest quintile of the Brazilian population used a public establishment (with 86% using a SUS establishment) in their last visit, compared to only 23% of the richest Brazilians (20% using the SUS) [53].

2 Throughout this paper, we use the concepts privatization, private sector involvement in the health system, and privately managed health care/services almost interchangeably. However, it should be noted that “privatization” is the broadest concept, which includes “private sector involvement in the health system,” and “privately managed health services” is, in turn, one type of private sector involvement.

3 The widespread availability of dental equipment is in part due to the 2004 implementation of the Brasil Sorridente (Brazil Smiling) program, the current National Dental Health Policy initiated to improve dental health in Brazil.

4 Recent figures show that the dental equipment sector is the most successful of all Brazilian medical device manufacturers. Although Brazil is the largest medical equipment market in Latin America, the majority of medical technology is imported. However, the dental equipment sector is the only device manufacturer that has seen a foreign trade surplus [54].

5 During the 2012 mayoral elections in São Paulo, the PT candidate Fernando Haddad accused the PSDB Candidate José Serra of having privatization “running through his veins” and of intending to privatize 25% of SUS beds [28].
